# Controlled study on Gamma nail and proximal femoral locking plate for unstable intertrochanteric femoral fractures with broken lateral wall

**DOI:** 10.1038/s41598-018-28898-6

**Published:** 2018-07-24

**Authors:** Lei Han, Jing-jing Liu, Yun-gen Hu, Ren-fu Quan, Wei-li Fang, Bo Jin, Wei-long Lin

**Affiliations:** 1 Department of Orthopaedics, XiaoshanTraditional Chinese Medical Hospital, Hangzhou, 311201 China; 2 Department of rheumatism and immunology, the Third Affiliated Hospital of Zhejiang Chinese Medical University, Hangzhou, 311201 China; 3 Department of Orthopaedics, Lishui Hospital of Traditional Chinese Medicine of Zhejiang, Lishui, 323000 China

## Abstract

The gamma nail and proximal femoral locking plate (PFLP) are both used for fractures. A controlled study was performed to determine the optimal implant. To assess and analyze the clinical effects of gamma nails and PFLPs for patients with unstable intertrochanteric femoral fractures, specifically with broken lateral walls. Thirty-six patients with unstable intertrochanteric femoral fractures and broken lateral walls were treated with gamma nails or PFLPs and retrospectively studied. The clinical data were compared. Duration of surgery and early full weight-bearing time were significantly longer in the PFLP group compared to the gamma nail group (P < 0.05). However, intraoperative fluoroscopy frequency and total blood loss in the PFLP group were significantly less than those in the gamma nail group (P < 0.05). No significant differences were found in hospitalized days, Parker Palmer mobility scores, Harris hip scores, and complications between the two groups. No difference in hip-functional recovery was found between the gamma nail group and the PFLP group, indicating that both the gamma nail and PFLP were effective for unstable intertrochanteric femoral fractures with a broken lateral wall. However, early weight bearing on the fractures was not encouraged in patients treated with PFLP.

## Introduction

Intertrochanteric femoral fractures occur between the greater and lesser trochanters and are commonly observed in the elderly^1^. Currently, internal fixation devices for treating unstable intertrochanteric femoral fractures are classified into intramedullary fixation and extramedullary fixation devices, both of which show advantages and disadvantages^2,3^.

The gamma nail is a classic intramedullary fixation device for treatment of intertrochanteric fractures that was developed by combining the sliding hip screw and intramedullary nail system^4^. The primary nail is placed in the intra medulla cavity, creating central fixation^5^. Several advantages of the gamma nail have been proposed, such as its application to the femoral head and neck and femoral shaft to increase stability at fracture sites and to promote healing^6^. In addition, its fixed application through the medulla cavity allows for early functional exercise and full weight-bearing of the affected legs. The standardized surgical technique for implementing gamma nails is accessible for surgeons, and the duration of operation is short, resulting in small operative wounds^7^. However, it is unsuitable for patients with thin or occlusive medullary cavities, very large anterior arches in femur shafts, obvious osteoporosis, and those with the risk of fat embolism^8^. The complications of the gamma nail include aggravated intertrochanteric fractures and stress fractures of the distal femoral shaft during the insertion of the main nail, in addition to breakage of the main nail, unsuitable lengths and locations of the lag screw, and the malunion of fractures are other complications that can occur^9^.

The proximal femoral locking plate (PFLP) is an extramedullary fixation device designed for fractures in the proximal femoral region, with an internal fixation trestle made of several locking screws that provides very good angular stability^10^. PFLPs are sclerotin independent for fracture fixation, which is beneficial for patients with osteoporosis, and comply with biological osteosynthesis, for which stability does not originate in friction between the plate and bone, resulting in reduced stress on the bone^11^. With the proximal plate matched to the greater trochanter, up to six fixed points can be used in the proximal femur, with five screws supporting the femoral neck and head and one targeting the calcar femorale. Fixation at multiple points maximizes resistance to rotation and prevents re-injury of the lateral femoral wall. The swollen top of the plate also has a gathering effect on comminuted fractures that enhance the lateral wall structure in the greater trochanter^12^. However, as an extramedullary fixation device, the PFLP should follow the tension band principles, which requires the integrity of the support structure at the posterior inner-side trochanter^13^. Reduced fixation or delayed weight bearing on the fracture must occur for dislocated lesser trochanters; otherwise, complications, including screw breakage and coxa vara, are more likely to occur^14^.

The lateral trochanteric wall is a key factor in lateral wall fractures that affects the stability of intertrochanteric fractures^15^. When two tangents are drawn separately along the superior and inferior of the femoral neck, the part of the lateral femoral cortex between the two tangents is defined as the lateral femoral wall^16^. This area is primarily cancellous bone, which is vulnerable to iatrogenic injury during surgery. Previous studies have confirmed that fracture of the lateral trochanteric wall was the primary independent predictor of reoperation^17^, and intertrochanteric femoral fractures are often treated with extramedullary fixation devices, such as dynamic hip screws, or intramedullary fixation devices, such as the gamma nail. The PFLP was developed with improved function for maintaining stability and avoiding rotation of the fracture sites, becoming an effective method for treating intertrochanteric femoral fractures^18^. To assess and analyze the clinical effects of gamma nails and PFLPs on patients with unstable intertrochanteric femoral fractures, specifically with broken lateral walls, this retrospective case-control study was conducted to determine the optimal implant for this type of fracture.

## Materials and Methods

### Subjects

This retrospective study was approved by the Ethics Committee of the Xiaoshan Traditional Chinese Medical Hospital and performed in accordance with the Helsinki Declaration. All patients agreed to participate and provided written informed consent prior to treatment. The inclusion criteria were patients over 18 years of age with fresh and closed fractures that had occurred within three weeks when they were enrolled in this study. Patients with unstable intertrochanteric femoral fractures with broken lateral walls were identified by preoperative computed tomography (CT) scans, and the follow-up time was 14–24 months.

Thirty-six patients were enrolled in the study between January 2014 and January 2016 and were assigned to two treatment groups: the gamma nail group and the PFLP group. The gamma nail group included 20 patients (11 males and 9 females, with a mean age of 58.4 ± 14.2 years, ranging from 29–88 years). The PFLP group included 16 patients (6 males and 10 females, with a mean age of 56.5 ± 12.4 years, ranging from 27–86 years).

Routine skin traction was required before each operation, and early operation was performed for patients who were eligible for principle anesthesia. All patients were examined using CT scans before each operation. For patients with severe comminuted lateral walls, thin or occlusive medullary cavities, or very large anterior arches in the femur shaft, the operation of intramedullary nails was difficult, and PFLPs were a better alternative.

### Surgical techniques

**Figure 1:**
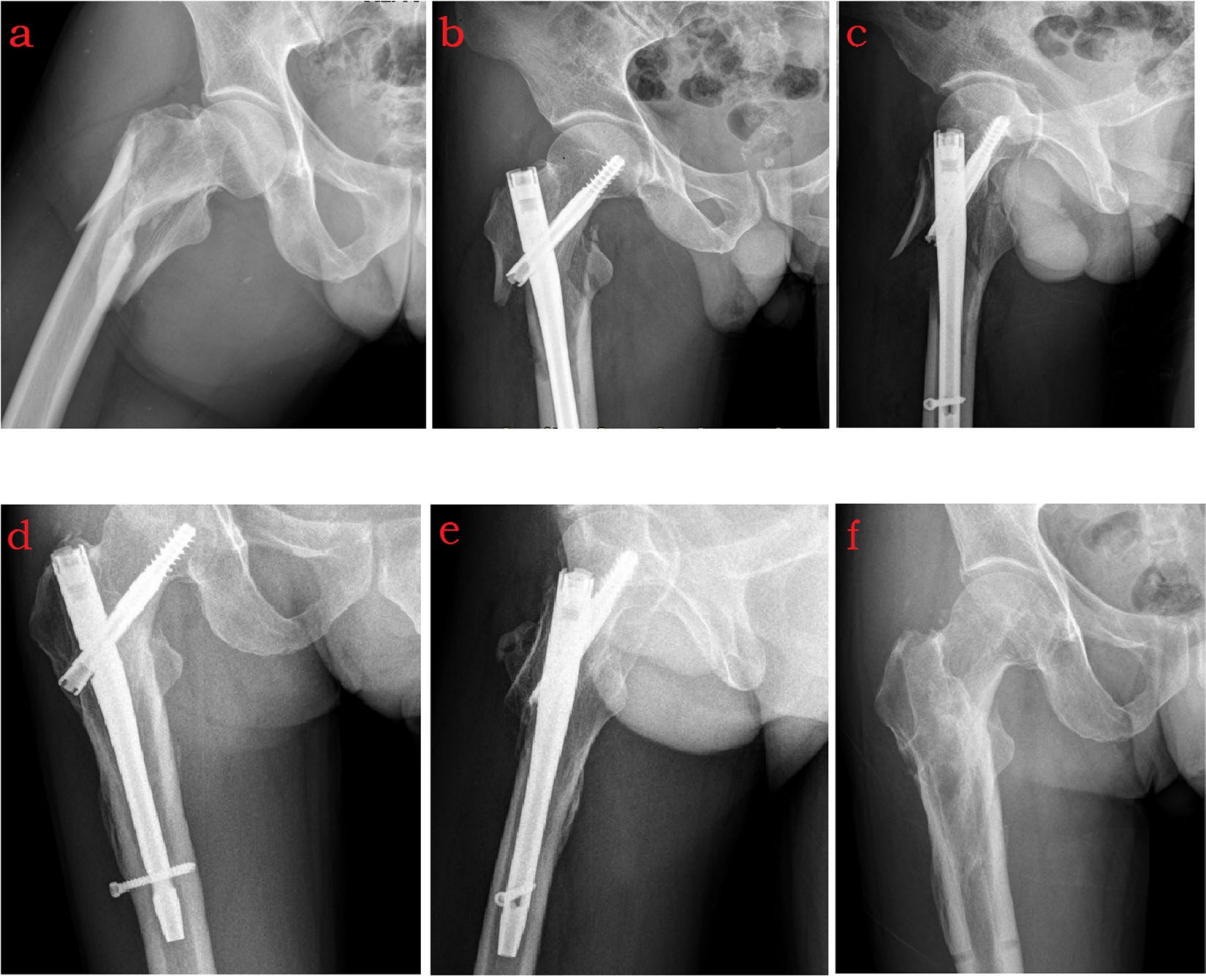
A 38-year-old male with right intertrochanteric fracture fixed with Gamma nail. (**a**) Radiograph of pelvis showed the fracture of right intertrochanteric, with broken lateral wall. (**b**,**c**) Anteroposterior and oblique plain film of right hip taken 2 weeks after surgery showed significant lateral wall defect. (**d**,**e**) Fracture healing was showed 6 months after surgery.

For gamma nail (Stryker Company, Gamma 3 Nail) operation, patients were placed in supine positions on the fracture table, and traction reduction was performed under C-arm fluoroscopic monitoring. After routine disinfection of the injured limb, a longitudinal incision of about 5 cm was made over the far end of the greater trochanter. A guide pin was then inserted into the medullary cavity along the end of the greater trochanter. After reaming along the guide pin, the gamma nail was inserted, and the guide pin was pulled out. Guided by the sighting device, the guide pin was inserted into the neck of the femur, at a distance of about 1 cm between the head-end of the guide pin and the articular surface of the femoral head. After the length of the head nail was determined, the core drill was used for reaming, and a head nail of suitable length was screwed into the hole. Finally, the distal nail was inserted, based on guidance from the sighting device, and the proximal end cap was then screwed into (Fig. 1).

**Figure 2:**
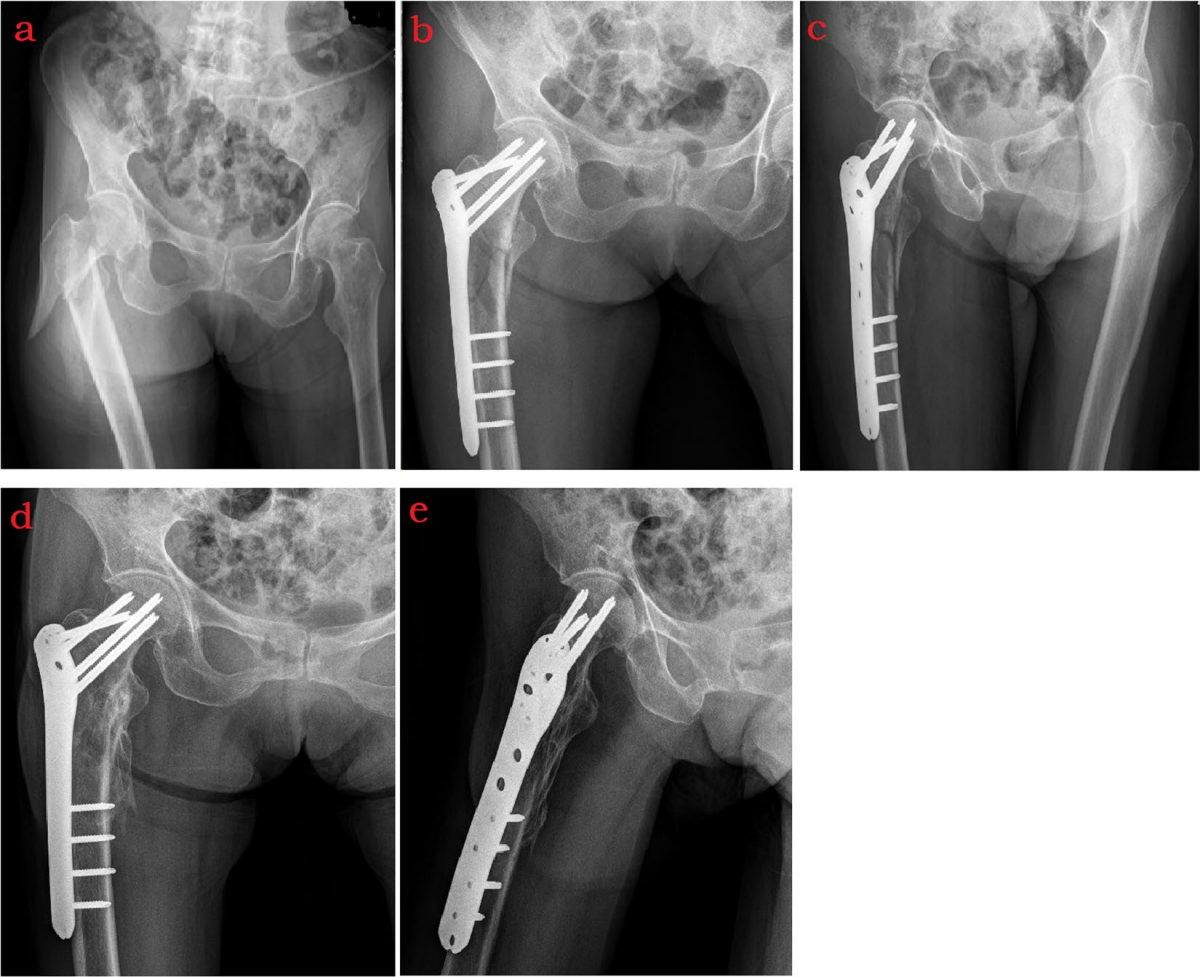
A 81-year-old female with right intertrochanteric fracture treated by proximal femoral locking plate (PFLP). (**a**) Radiograph of pelvis showed fracture of right intertrochanteric, with broken lateral wall. (**b**) Anteroposterior plain film of right hip taken 3 days after surgery showed satisfactory reduction of fracture. (**c**) Oblique plain film of right hip taken 3 days after surgery showed well aligned fracture, with stable internal fixation. (**d**,**e**) Fracture healing was showed in anteroposterior and oblique plain film of right hip taken 8 months after surgery.

For PFLP (Smith&Nephew company) surgery, patients were placed in a supine position on the X-ray radiolucent traction table, and limited open reduction was used for patients when closed reduction was unsuccessful. The fascia lata and vastus lateralis were incised to visualize the femoral trochanter, and the vastus lateralis was stretched toward its anterior inferior, without stripping the periosteum, to reset the fracture’s broken end. The collodiaphyseal and anteversion angles were recovered, and a PFLP of an appropriate length was inserted along the outside surface of the femur, ensuring that the plate and the proximal femur were in good contact. With the position of the plate confirmed by C-arm fluoroscopy, locking screws were inserted at four to six points, based on the guide handle at both the distal and proximal ends of the fracture. The postoperative fracture reduction and internal fixation position were re-examined by X-ray fluoroscopy (Fig. 2).

### Postoperative management

Patients in both groups received conventional intravenous drips of antibiotics for three days, and 24 hours after operation, patients were required to perform lengthening contraction exercises of the quadriceps femoris and flexion and extension activities involving the hip joints, under guidance of the surgeon. Low molecular weight heparin was used from the first day in hospital to two weeks after the operation to prevent thrombosis in the lower extremities.

One week after operation, patients with gamma nails (including elderly patients with obvious osteoporosis) started partial weight bearing movement (i.e., 15% of body weight), gradually transitioning to full weight-bearing movement by six to eight weeks after operation, depending on the fracture type and fracture healing. Patients with PFLPs started partial weight-bearing movement (again, 15% of body weight) after callus was identified using X-ray fluoroscopy (i.e., three weeks after operation), gradually transitioning to full weight-bearing movement depending on the fracture type and fracture healing. For partial weight bearing, patients were supported by a walker, double crutches, or a single crutch, with a bathroom scale laid below the affected limb to guarantee 15% body weight.

### Therapeutic evaluation and follow-up

The duration of surgery, intraoperative fluoroscopy frequency, total volume of intraoperative and hidden blood loss, and hospitalized days were recorded for all patients in both groups. Hidden blood loss was calculated using the Gross equation^19^, according to the height, body weight, and alteration of hematocrit of each patient both before and after operation. The patients were followed up once a month for six months after the operation and then once every three months for six more months.

Radiologic evaluations were conducted to estimate the condition of fracture healing, coxa vara malformation, and internal fixation failure. Specifically, front and lateral radiographs were taken once a month for six months after operation, and X-ray films were taken once every three months for a further six months. Hip-joint function was evaluated based on the Parker Palmer mobility score (PPMS)^20^ (Table 1) and Harris hip score (HHS)^21^. The hip joint function was evaluated by HHS for four aspects: pain, function, deformity, and range of motion, with a full mark being 100. Scores between 90 and 100 were excellent, scores between 80 and 89 were good, scores between 70 and 79 were fair, and scores less than 70 indicated poor hip-joint function.

**Table 1:** Parker Palmer mobility score (PPMS).

Walking	Without difficulty	With a walker	Need help from others	Disability
Outdoor	3	2	1	0
Indoor	3	2	1	0
Ability of daily living	3	2	1	0

### Statistical analysis

The SPSS 14.0 software was used for data analysis. Patients’ ages, durations of surgery, intraoperative fluoroscopy frequency, hospitalized days, and HHSs were normally distributed data, represented as mean ± SD. The differences between the two groups were analyzed using a t-test. In contrast, blood loss and early full weight-bearing time were abnormally distributed data, represented as medians and analyzed using a Mann–Whitney test. The Chi-square (X2) test was used to analyze enumeration data, such as gender, fracture type, and complications. A value of P < 0.05 was taken as statistically significant.

## Results

### Comparison of basic clinical data between the two groups before operation

As shown in Supplemental Table 1, no significant differences in preoperative clinical data, such as age, gender, and fracture type, were found between the gamma nail and PFLP groups.

### Comparison of surgery data between the two groups

The results showed that the duration of surgery in the PFLP group (70.5 ± 20.2 min) was significantly longer than in the gamma nail group (60.6 ± 20.8 min; P < 0.05). Intraoperative fluoroscopy frequency was significantly less in the PFLP group (5.2 ± 3.4 times) compared with the gamma nail group (8.8 ± 4.8 times; P < 0.05). In addition, total blood loss, including intraoperative and hidden blood loss, was significantly less in the PFLP group (60~600 ml) compared to the gamma nail group (300~600 ml; P < 0.05). However, there was no significant difference in hospitalized days between the two groups (Supplemental Table 2).

### Comparison of postoperative recovery between the two groups

The patients were followed up for 14–24 months with an average time of 18.5 months after operation. Three cases resulted in pulmonary infection, which was cured using anti-infective therapy. The healing time of the fractures was 20.6 ± 3.6 weeks among PFLP patients and 18.8 ± 3.8 weeks among gamma nail patients, showing no significant difference between the two groups. Early full weight bearing among PFLP patients was significantly later than among gamma nail patients (P < 0.05). No significant difference in PPMS was found between the PFLP group (6.84 ± 1.48) and the gamma nail group (7.28 ± 1.72) at the final follow-up. The HHSs of PFLP patients was 83.18 ± 12.16 and among gamma nail patients was 86.22 ± 11.28, and no significant difference was found between the two groups (Supplemental Table 3).

### Comparison of complications between the two groups

In the gamma nail group, breakage of the internal fixation was found in one case, for which internal fixation was removed after fracture union. Another case resulted in femoral head necrosis, and hip replacement surgery was performed eight months after operation. In the PFLP group, two patients suffered from coxa vara and were treated with traction in bed for six months until fracture union, without further treatment. Therefore, there was no significant difference in the incidence of postoperative complications between the two groups (Supplemental Table 3).

## Discussion

Both gamma nails and PFLPs were used to treat unstable intertrochanteric femoral fractures with broken lateral walls, and the duration of surgery, intraoperative fluoroscopy frequency, total blood loss, hospitalized days, postoperative complications, and limb functional recovery were analyzed between two implant groups. The results indicated that both gamma nails and PFLPs are effective for unstable intertrochanteric femoral fractures with broken lateral walls for hip functional recovery. Compared to the PFLP, the gamma nail had the advantages of shorter duration of surgery and earlier full weight-bearing time; however, disadvantages were higher intraoperative fluoroscopy frequency and total blood loss. The transition from partial weight bearing to full weight bearing was completed gradually depending on the fracture type and fracture healing. With a smaller surgical incision and better self-perception, patients treated with gamma nails started partial weight bearing one week after operation, while patients with PFLPs began partial weight bearing after callus was identified.

This study confirms that gamma nails, an intramedullary fixation device, are effective for unstable intertrochanteric femoral fractures with broken lateral walls. However, in cases with free bone fragments in the greater trochanter, the lateral trochanteric wall shatters and cleavage occurs in the coronal plane in the trochanteric site; thus, the intramedullary nail might cause further lateral wall damage, aggravating the instability of the wall^22^. In this study, two patients (10%) with gamma nails developed complications, including screw breakage and femoral head necrosis, which was a higher complication occurrence compared to a previous study^23^; this might be the result of the small sample size. Compared to intramedullary nails, PFLPs has been deemed to be a better choice for fractures with an AO (AO Foundation/Orthopaedic Trauma Association [AO/OTA] classification) type above 31-A3.2 accompanied by a broken lateral wall^12^. This study demonstrated that the PFLP showed no advantages in duration of surgery and early full weight-bearing over gamma nail, which may be further investigated with bigger sample size. In addition, the PFLP had the advantage of lower total blood loss, including intraoperative blood loss and hidden blood loss. In clinics, the hidden blood loss for gamma nails was usually neglected because of small amounts of intraoperative blood loss. Even with a minimally invasive surgery, the gamma nail is more likely to result in hidden blood loss and postoperative anemia^24,25^, which may be related to medullary reaming, the opened medullary cavity, and blood sinus in the proximal femoral cancellous bone.

The PFLP is an extramedullary fixation device that is used with increasing frequency because it offers improved function for maintaining stability and preventing rotation of the implant^26^. The swollen top of the plate plays a crucial role in gathering comminuted fractures, reinforcing the structure of the lateral wall at the same time. The PFLP was specifically designed for fractures in the proximal femoral region to increase early mobilization of patients by placing more screws into the proximal femur at different angles^27^, which is theoretically superior to the intramedullary nail. The PFLP used in this study was designed for fractures in the proximal femur and recommended by its producer for the treatment of unstable intertrochanteric femoral fractures. With six fixed points at the proximal femur, the PFLP maximized rotation resistance, preventing re-injury of the lateral femoral wall^28^. Especially for patients with severe comminuted lateral walls, for which it was difficult to use intramedullary nails, the PFLP was superior to the gamma nail.

The importance of the lateral trochanteric wall was highlighted in the present study. Previously, the stability of intertrochanteric femoral fractures largely relied on the lesser trochanter and the degree of the comminuted fracture of the posterior medial wall. The lateral trochanteric wall is likely to suffer from secondary damage during the insertion of intramedullary fixation devices, resulting in a high incidence of lateral wall blowout after operation^29^. Regardless of whether fractures of the lateral wall exist before operation, iatrogenic blowout is likely to occur in the lateral wall after operation. In one study, broken lateral walls occurred in 24 patients with pertrochanteric fractures treated by internal fixation, confirming the importance of the integrity of the lateral trochanteric wall in unstable fractures^15^. In the current study, cortical cleavage of the lateral wall occurred in three cases of patients treated with gamma nails, in which gamma nail failed to maintain stability of the femur; these nails were replaced by PFLPs. Thus, as an optimal choice for the treatment of unstable intertrochanteric femoral fractures with broken lateral walls, the PFLP was more beneficial in protecting broken lateral walls compared to the gamma nail.

This was a controlled study with certain limitations. The sample size was relatively small and the length of follow-up was relatively short. Improvement of these conditions in future studies may provide more information for validating the results presented here.

In conclusion, gamma nails and PFLPs were both effective for unstable intertrochanteric femoral fractures with broken lateral walls for hip-functional recovery. The PFLP was more effective in severe comminuted fractures and more beneficial for protecting the broken lateral wall, compared to the gamma nail; however, early weight bearing was discouraged among patients treated with PFLPs.

## Electronic supplementary material

Supplementary Information
