# Supplementary material for: Controlled study on Gamma nail and proximal femoral locking plate for unstable intertrochanteric femoral fractures with broken lateral wall
Source: Sci Rep. 2018 Jul 24;8:11114. doi: 10.1038/s41598-018-28898-6 (PMC6057971; doi:10.1038/s41598-018-28898-6)
Supplement: Supplementary file 1 — Supplementary Information [file 41598_2018_28898_MOESM1_ESM.pdf]

# Controlled study on Gamma nail and proximal femoral locking plate for unstable intertrochanteric femoral fractures with broken lateral wall

Lei Han[1]*, Yun-gen Hu[1], Ren-fu Quan[1], Wei-li Fang[1], Bo Jin[1]

[1]Department of Orthopaedics, Xiaoshan Traditional Chinese Medical Hospital, Hangzhou, 311201, China

***Corresponding author:** Lei Han

**Mailing address:** Department of Orthopaedics, Xiaoshan Traditional Chinese Medical Hospital, Hangzhou, 311201, China

**Address:** Yucai Road No. 152, Hangzhou, Zhejiang, 311201, China

**Running title:** Proximal femoral locking plate for femoral fractures

**Correspondence to：leihan589@163.com**

**Supplemental Table 1. Comparison of basic clinical data of the two groups before the operation**

| Groups | Cases (n) | Ages (years) | Gender | | Fracture type | | | | |
|---|---|---|---|---|---|---|---|---|---|
| | | | Male | Female | 31A2.2 | A2.3 | 31A3.1 | 3.2 | 3.3 |
| Gamma nail | 20 | 58.4±14.2 | 11 | 9 | 4 | 6 | 6 | 3 | 1 |
| PFLP | 16 | 56.5±12.4 | 6 | 10 | 2 | 3 | 4 | 5 | 2 |
| *t*/ $x^2$ | | *t*=1.248 | $x^2$=0.242 | | $x^2$=0.862 | | $x^2$= 1.869 | | |
| *P* value | | 0.264 | 0.728 | | 0.472 | | 0.076 | | |

PFLP, proximal femoral locking plate.

**Supplemental Table 2. Comparison of surgery data in the two groups**

| Groups | Cases (n) | Duration of surgery (min) | Total blood loss [ml, $M(Q_1 \sim Q_3)$] | Fluoroscopy frequency (times) | Hospitalized days |
|---|---|---|---|---|---|
| Gamma nail | 20 | 60.6±20.8 | 350 (300~600) | 8.8±4.8 | 12.6±5.8 |
| PFLP | 16 | 70.5±20.2 | 150 (60~600) | 5.2±3.4 | 15.1±6.6 |
| *t*/ $x^2$ | | 0.532 | 0.241 | 0.481 | 1.765 |
| *P* value | | 0.026 | 0.012 | 0.018 | 0.882 |

PFLP, proximal femoral locking plate.

**Supplemental Table 3. Comparison of postoperative recovery in the two groups**

| | Gamma nail | PFLP | *P* value |
|---|---|---|---|
| Early full weight bearing [months, M(Q1-Q3)] | 2 (1~6) | 2 (3~10) | 0.034 |
| Healing time of fracture (weeks) | 18.8 ±3.8 | 20.6 ±3.6 | 0.328 |
| PPMS | 7.28 ±1.72 | 6.84 ±1.48 | 0.142 |
| HSS | 86.22 ±11.28 | 83.18 ±12.16 | 0.264 |
| Excellent | 5 | 3 | |
| Good | 6 | 6 | |
| Fair | 7 | 6 | |
| Bad | 2 | 1 | |
| Incidence rate of complications | 10% (2/20) | 12.5% (2/16) | 0.253 |

PFLP, proximal femoral locking plate.
